# AI and the democratization of knowledge

**DOI:** 10.1038/s41597-024-03099-1

**Published:** 2024-03-05

**Authors:** Christophe Dessimoz, Paul D. Thomas

**Affiliations:** 1 Swiss Institute of Bioinformatics, Lausanne, Switzerland; 2 Department of Computational Biology, University of Lausanne, Lausanne, Switzerland; 3 Department of Population and Public Health Sciences, University of Southern California, Los Angeles, USA

**Keywords:** Research data, Funding, Scientific community, Computational biology and bioinformatics

## Abstract

The solution of the longstanding “protein folding problem” in 2021 showcased the transformative capabilities of AI in advancing the biomedical sciences. AI was characterized as successfully learning from protein structure *data*, which then spurred a more general call for AI-ready datasets to drive forward medical research. Here, we argue that it is the broad availability of *knowledge*, not just data, that is required to fuel further advances in AI in the scientific domain. This represents a quantum leap in a trend toward *knowledge democratization* that had already been developing in the biomedical sciences: knowledge is no longer primarily applied by specialists in a sub-field of biomedicine, but rather multidisciplinary teams, diverse biomedical research programs, and now machine learning. The development and application of explicit knowledge representations underpinning democratization is becoming a core scientific activity, and more investment in this activity is required if we are to achieve the promise of AI.

At the heart of empirical science is data, and its transformation into knowledge. Historically, with few exceptions, any given datum was interesting only to the specialist in a narrow scientific domain. However, with increasing specialization and mounting volumes of data, science relies increasingly on cross-disciplinary interactions, and no area more so than biomedical research. The demand for data democratization—i.e., making data understandable and useful to non-specialists—was a driving force behind the open science movement^1^ and the advent of biocuration in the 1990s and its development for the following quarter century. The continuation of this trend has also motivated recent calls for the FAIR (findable, accessible, interoperable, reusable) principles of data sharing^2^.

Today, the growing power of computational algorithms in the scientific process, including AI, have brought this trend into a completely new phase, as democratization now means broadly usable by both humans and machines. **AI is “data-hungry”; untrained, it is the ultimate non-specialist**. The FAIR principles are a necessary step in feeding AI, but they may not always be sufficient. It is well known that the preparation of data suitable for AI can often be the most time-consuming part of machine learning^3^. But what does it mean to prepare suitable data for AI? We suggest that it often means transforming the data into a form that can more accurately be thought of as *knowledge*, rather than data. More precisely, we argue that scientific knowledge, i.e. models of actual physical entities and processes, should be represented in standardized, clearly documented formats that allow the knowledge to be correctly interpreted and used by a broad community comprising both non-experts and AI (Fig. 1). Thus, the transformations of data to knowledge should be made with the explicit aim of democratizing the data and knowledge, and we should always ask of any data set: which transformations are required to produce a knowledge representation that is usable by as large a community as possible, including machines?

**Fig. 1:**
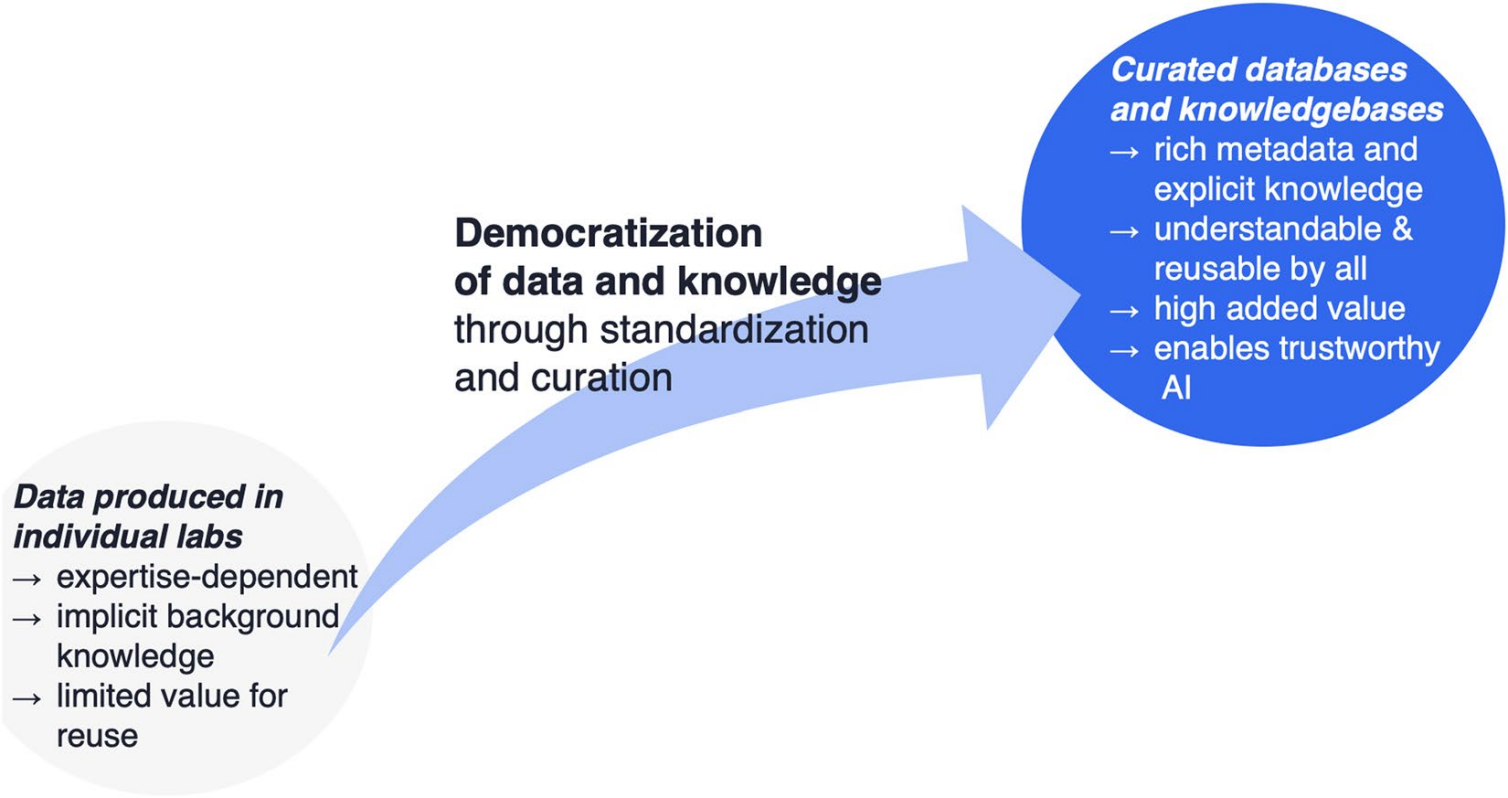
The need for democratization of data and knowledge. For data to become broadly valuable, it must be transformed into a form that can be correctly interpreted and used by a broad community comprising both non-experts and AI.

## Training AI to solve the protein folding problem depended on knowledge

The recent revolutionary AI breakthrough by AlphaFold2^4^ and RoseTTAFold^5^ toward solving the long-standing protein folding problem was an important example of AI making a major impact in the biological sciences. It is also a good example of an important kind of problem that AI can solve in principle: generalize from a limited number of “training examples” to a potentially unlimited number of cases. The protein folding problem has long been considered one of the holy grails of biomedical research. DNA sequencing technology has enabled us to determine the “primary structure,” or sequence of amino acid residues, for millions of different proteins, and more every day. But it is the three-dimensional, “tertiary structure” of the protein that determines how it acts *in vivo* to perform the chemical reactions and interactions that sustain life and ultimately determine health and disease. Determining a protein structure by experiment is costly and often intractable, preventing us from applying these technologies to more than a small fraction of proteins. Solving the protein folding problem – using sequence information alone to find the 3D structure of any and all proteins – opens a path to revolutionize biomedical research and accelerate the discovery of drugs (most of which work by directly interacting with a folded protein).

The AI training examples used by AlphaFold2 and RoseTTAFold are often referred to as “protein structure data,” but we suggest that this usage of the term is somewhat misleading to non-experts. As commonly understood, data corresponds to an observation, often measurable. In the case of protein structures, the data are X-ray diffraction patterns, cryo-electron microscopy images or magnetic resonance spectra. Through an extremely complex process, these data are used to construct a three-dimensional *model* of a protein structure. It was the three dimensional models, not the raw data itself, that was used to train AI algorithms.

**Fig. 2:**
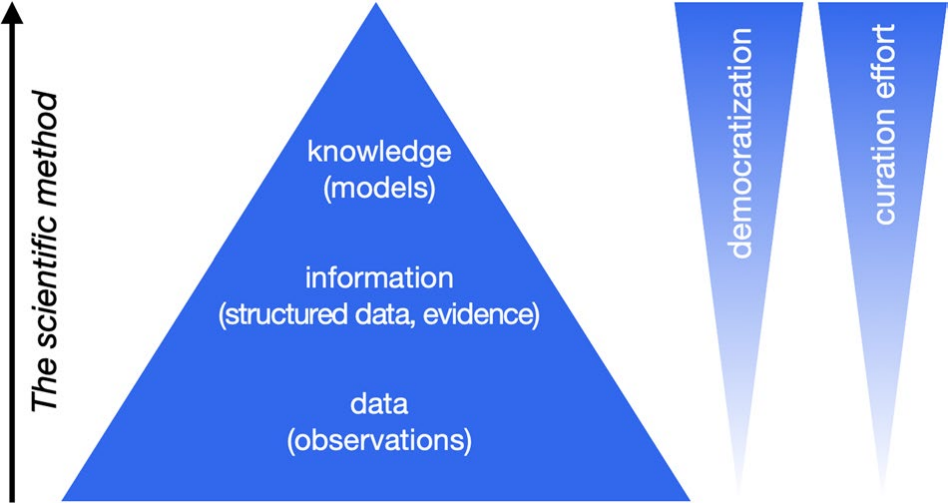
The data-information-knowledge hierarchy in empirical sciences and the path to democratization. Increasing democratization requires additional effort to transform toward consistent, explicit knowledge models, which for complex models requires extensive curation of training sets sufficient for AI.

This example suggests an operational definition of *knowledge* as understood by scientists: it is similar to the concept of a scientific model, i.e. a formal representation of a physical entity or process, supported by evidence and testable by further experiment and observation. This definition is consistent with the range of definitions of knowledge that have emerged in the fields of data science and knowledge representation, as originally proposed in the data-information-knowledge hierarchy^6^ and reviewed by Rowley^7^, who suggests a consensus definition of knowledge as “a mix of information, understanding, capability, experience, skills and value” (Fig. 2). This definition would not include raw data, or the structured, organized or applied data that usually constitutes “information” (or evidence^8^), but it would seem to fit the usage of the word “model” in science.

## How do we achieve democratization of data and knowledge?

In an individual lab or a community of specialists, local expertise is sufficient to ensure the data integrity, consistency and background knowledge to make full use of a dataset. Democratization implies that such local expertise can no longer be assumed. In simple terms, we suggest that the key to democratization is transforming or augmenting the data into a consistent, formal knowledge representation (Fig. 2). In the knowledge management literature, there has been extensive discussion of explicit versus implicit knowledge, with the main distinction being that “explicit knowledge is codified and recorded, and as such is designed for sharing”^7^.

For relatively *simple*, *routine* kinds of data or knowledge, i.e. for which the underlying data processing or knowledge representation is well-established, democratization can be largely achieved by standardization. For instance, the knowledge of nucleotide sequences (which we suggest can be considered a model of the physical covalent bonds linking successive nucleotides in a DNA sequence) are derived from raw measurements of fluorescence peaks (or other raw data, depending on the specific sequencing technology), but the data processing steps are well-established and can be considered to be routine. IUPAC defines a universally adopted standard for representing nucleotide and protein sequence data. The IUPAC code for nucleotide and protein sequences is the universally adopted standard for representing sequence knowledge, and nobody needs to worry whether L stands for Leucine or Lysine (it’s the former). The standardization of data processing and representation is sufficient to automatically produce consistent knowledge. Likewise, for various kinds of high-throughput omics data, various minimum information standards have been established by the relevant communities. Deposition to specific repositories, such as GenBank or the European Nucleotide Archive for genetic sequences, is part of this standardized transformation process, as it enforces some consistency and metadata collection upon data deposition. And by requiring standardized data deposition in the first place, journals can also contribute toward the democratization of simple, routine knowledge. Note that the need to provide metadata and undergo consistency checks, also holds for raw data repositories, such as the Electron Microscopy Public Image Archive for raw images underpinning 3D cryo-EM maps and tomograms^9^.

But standardization does not work for all kinds of data or knowledge. First, it takes time and effort to establish new standards, which tend to lag behind technological development. Second, to achieve wide adoption, standardized knowledge representations tend to capture the “common denominator,” which leaves out potentially valuable information. And third, once adopted, it can be difficult to evolve a standard. This lack of flexibility can stand in the way of scientific and technological advances.

For transformation of *emerging*, *complex*, or *unconventional* kinds of data or knowledge—particularly those arising from a new technology or methodology—a different approach is generally required. As described above, protein structure knowledge is an example of a complex transformation. To solve the protein folding problem, AI didn’t use raw data, nor did it scour the internet and journals for protein structure knowledge. It had a ready source of consistent, explicit models available in the Protein Data Bank (PDB)^10^, in a standard format for representing a three dimensional model of a protein. Achieving a broadly usable knowledge representation – a model expressed in terms of the coordinates of each atom in the protein along with critical metadata – depended on deep human expertise, both within the individual structure-determination laboratories, and within the PDB resource. First, biocuration experts at PDB adopted a detailed specification for how a protein structure model should be represented, called the macromolecular crystallographic information file (mmCIF/PDBx), which defined all model data and metadata in a way that is fully machine-readable. This specification is extensible and has been updated and expanded numerous times, most notably as new technologies such as NMR and cryo-EM have emerged. Second, PDB biocurators work together with each group of experimentalists who deposit a protein structure in PDB, to ensure it adheres to this rigorous specification as well as model quality standards. Biocurators at the PDB are the indispensable partners in the democratization process: without their effort and expertise, the use of protein structure data would be limited to specialists with detailed knowledge of macromolecular structure, biochemistry and structure-determination technology. Instead, the partnership between experimentalists and biocurators at the PDB has democratized 3D macromolecular structure data, providing the knowledge in a form that could be used in AI training.

The PDB is just one example of what we see as an emerging role in the biomedical scientific process, brought on by the complexity of biological systems and the sheer volumes of data: creating knowledge representations through synthesis and consensus. An arguably even more complex transformation of data to knowledge – and one with which we are highly experienced as leaders of the Gene Ontology and UniProt/Swiss-Prot knowledgebase projects – is the construction of models of how proteins function together in biochemical and signaling pathways, or to regulate processes such as the cell cycle. This type of knowledge modeling requires a consensus interpretation of multiple lines of evidence and sources of information and knowledge. Within an area of specialization, establishing this consensus is a familiar part of the scientific discourse—through the scientific literature, conferences, and exchanges among specialists. However, democratization to other communities, and especially to machines, requires a breadth of synthesis and consensus that goes beyond the traditional venues of conferences, literature reviews and textbooks. This crucial role has been filled by expert biocurators. Biocurators are trained in both data science–specifically the human and computer-readable representations of knowledge–and broad biomedical concepts that span multiple specialties.

The explicit encoding of knowledge in computer-readable format, i.e. making knowledge AI-ready, is essential for big data analysis and machine learning. This is an ongoing process. The evaluation of potential additions and revisions to a scientific model requires an understanding of how it fits with the existing model, as well as the current consensus in a specific area, which evolves as new experiments are performed, and new technologies are developed. Such tasks are at the heart of the scientific enterprise, and are unlikely to be replaced by machine learning in the near term; yet given enough high-quality positive and negative training examples, AI can greatly accelerate the process, with some successes in this area already reported^11^. Consequently, realizing the potential of computational methods such as machine learning has only increased the demands on biocuration.

## Call for action – sustaining biocuration as a catalyst of the AI revolution

All the above highlight the central role biocuration plays in democratizing data and knowledge, and thus in the AI revolution. What are the direct consequences then for biocurators, specialists, and funders?

Biocurators should be recognised as agents of democratization in the scientific enterprise. This role places them at a key position in the scientific process and in the application of AI. We acknowledge this turns on its head the popular connotation of curation as a niche activity. Rather, it is becoming critical to continued scientific progress, and requires a deep understanding of the relevant biological and technological domains, data science proficiency, and an ability to collaborate with both domain specialists and computer scientists.

Domain specialists need to see biocurators as partners in the scientific process, who help ensure their discoveries have a relevance and impact beyond their subfields. They will benefit from working with biocurators to ensure that knowledge in their subfields is faithfully encoded in the consensus models that will be broadly used by other researchers and machines.

Funders need to recognise that investments in biocuration are high return-on-investment, as they provide the path to democratization. For simple, and routine data which can be standardized, biocuration can be largely automated and is thus highly efficient. For emerging, complex or unconventional data and knowledge, biocuration is typically more labor intensive, but the costs are typically only a small fraction of the costs of generating them in the first place, while increasing their value many fold^12,13^. Thus, biocuration plays both a critical and cost-effective role in achieving democratization. Despite that, the potential of biocuration is yet to be fully realized, as funding of biocuration activities has not kept up with usage growth in the last decade. For instance, in that period NCBI’s budget for “informatics resources for biomedicine and health” has remained flat, while usage has grown almost threefold^14^. Community curation has been explored as a way to distribute the curation work, with mixed success^15–17^; but even resources with a substantial community curation rely on professional curators to ensure quality and consistency^18,19^.

Database and knowledgebase groups are already painfully aware of the limited nature of biocuration resources, but we suggest that explicitly considering democratization as the overarching goal can help to set priorities. For example, just as it should be, these resources are managed by scientists with expertise in the appropriate domain. As a result, there can often be an innate tendency to focus on serving experts like themselves, and not necessarily a broader community. We suggest that democratization can be used as a guiding principle for choosing which knowledge representations should be adopted, which data should be transformed, and which metadata are really the most valuable to include. Likewise, the goal of democratization could be used to balance data sharing requirements with the cost in time and resources they incur. Who is expected to benefit from the data and knowledge, and what representation of the knowledge, if any, will facilitate broad reuse?

In summary, AI requires more biocuration, targeted to the right areas, and not less. Recognizing and embracing this fact will help drive further gains in AI and scientific and medical progress in general.
